# Reactive Oxygen Species in Cancer Biology and Anticancer Therapy

**DOI:** 10.1155/2016/4197815

**Published:** 2016-10-03

**Authors:** Alexandr V. Bazhin, Pavel P. Philippov, Svetlana Karakhanova

**Affiliations:** ^1^Department of General, Visceral, and Transplantation Surgery, Ludwig-Maximilians-University Munich, 81377 Munich, Germany; ^2^Department of Cell Signalling, A. N. Belozersky Institute of Physico-Chemical Biology, Moscow State University, Moscow 119991, Russia; ^3^Surgical Research Section, University of Heidelberg, Heidelberg, Germany; ^4^Department of General, Visceral and Transplantation Surgery, University of Heidelberg, 69221 Heidelberg, Germany

Our understanding of reactive oxygen species (ROS)—a group of highly reactive chemicals containing oxygen—has changed in the last few years from ROS as just harmful substances to crucial intra- and extracellular messengers as well as important regulators controlling a wide spectrum of signaling pathways. Nevertheless, there are still many uninvestigated points and open questions regarding ROS, especially in pathophysiology. Delicately controlled ROS homeostasis is critical for maintaining normal cell functions and any disruption in the oxidation-antioxidation balance leads to oxidative stress associated with a wide spectrum of human disorders such as chronic inflammation, age-related diseases, and cancers. In health, the intracellular ROS level is tightly controlled by various antioxidants. In contrast, cancer cells have an abnormally high level of ROS due to an increased ROS production and/or impaired ROS detoxification that can damage intracellular macromolecules such as nucleic acids, proteins, and lipids. Elevated ROS production in cancer cells may result from an aberrant metabolic activity, mitochondrial dysfunction, disturbed cellular signaling, oncogene activity, and interaction with tumor infiltrating immune cells.

The ultimate purpose of this special issue is to publish high-quality research communications as well as review articles dedicated to the role of ROS in cancer biology, anticancer therapy, and related topics. Five articles published in this special issue are devoted to reactive oxygen species in cancer biology. Presently, it is rather well known that H_2_O_2_ has the opposite effects on cancer cell proliferation depending on its concentration and cancer type. G. Vilema-Enriquez et al. in their article “Molecular and Cellular Effects of Hydrogen Peroxide on Human Lung Cancer Cells: Potential Therapeutic Implications” review effects of hydrogen peroxide on human lung cancer. The authors discussed effects of H_2_O_2_ on migration and invasion, calcium release, and other molecular features of cancer cells. Furthermore, they describe the link between hydrogen peroxide and inflammation. Finally, the authors hypothesize that novel therapeutic approaches against lung cancer may be based on the use of H_2_O_2_. Y.-C. Hung et al. in their review “Roles of Reactive Oxygen Species in Anticancer Therapy with* Salvia miltiorrhiza *Bunge” deal with Danshen as a drug of the traditional Chinese medicine and provide a systematic review of its antioxidant capacity and potential anticancer effects. Moreover, they conclude that based on the existed preclinical data this drug may be pipelined in clinical trials. A research paper by W. Li et al. (“Hyperglycemia Promotes the Epithelial-Mesenchymal Transition of Pancreatic Cancer via Hydrogen Peroxide”) deals with hyperglycemia in pancreatic cancer cells. The authors succeeded in finding the link between hyperglycemia and epithelial-mesenchymal transition through the production of hydrogen peroxide. Another research report on breast cancer of D. M. Badr et al. (“The Combination of *α*-Tocopheryl Succinate and Sodium Selenite on Breast Cancer: A Merit or a Demerit?”) shows in vitro and in vivo that sodium selenite antagonizes effects of *α*-tocopheryl succinate on apoptosis induction in cancer cells via inhibition of oxidative stress. An intriguing review came from France, authored by M. Assi and A. Rébillard, and was devoted to the problem of cachexia in cancer patients (“The Janus-Faced Role of Antioxidants in Cancer Cachexia: New Insights on the Established Concepts”). As regulators of catabolic pathways ROS are involved in muscle atrophy in cachectic cancer patients, the authors summarize and discuss contradictory data on the effects of antioxidants in such patients.

The next topic highlighted in this issue is devoted to ROS in tumor immunology. A review by X. Chen et al. (“Reactive Oxygen Species Regulate T Cell Immune Response in the Tumor Microenvironment”) gives readers an overview of ROS in the tumor microenvironment and especially in the tumor-induced immunosuppression. The authors, based on improvement of anticancer T cell response, consider an antioxidant treatment as a promising option for cancer therapy. A. Scala et al. in their research article “Alterations in Red Blood Cell Functionality Induced by an Indole Scaffold Containing a Y-Iminodiketo Moiety: Potential Antiproliferative Conditions” deal with a prediction of the antiproliferative effects of heterocyclic scaffolds, which could be important for development of new therapeutic approaches against cancer.

A research article by M. Weniger et al. (“The Analgesic Effect of the Mitochondria-Targeted Antioxidant SkQ1 in Pancreatic Inflammation”) considers pancreatitis as a main risk factor for pancreatic cancer. The authors show an unexpected analgesic effect of the new antioxidant SkQ1 during pancreatic inflammation. The last article from this issue deals with the oxidative stress in cancer-prone diseases in pediatric age. S. Perrone et al. in “Oxidative Stress in Cancer-Prone Genetic Diseases in Pediatric Age: The Role of Mitochondrial Dysfunction” review recent literature on such diseases and discuss molecular mechanisms of oxidative stress associated with mitochondrial dysfunction. They conclude that mitochondria-targeted medicines could be applied into the clinics to improve the quality of life of patients with cancer-prone genetic diseases.

Summarizing, the wide spectrum of review and research articles presented in this issue provides recent interesting data on ROS in the context of cancer biology and anticancer therapy.

*Alexandr V. Bazhin* *Alexandr V. Bazhin* *Pavel P. Philippov* *Pavel P. Philippov* *Svetlana Karakhanova* *Svetlana Karakhanova*

